# Good Health by Right Living

**Published:** 1888-11

**Authors:** 


### GOOD HEALTH BY RIGHT LIVING.

NEWARK N. J., Sept. 18, 1888.

Editor of HALL'S JOURNAL OF HEALTH.

*Dear Sirs;*—Will you be so kind as to inform me whether or not meat is a necessary article in a working man's daily food, *necessary* to produce the muscle needed for labor, and does not *wheat bread* made of that flour which is made of the *whole* kernel of wheat, sometimes called *health bread* contain *all the necessary elements* to produce muscle? also, oat meal and other kinds of grain?

Please answer this question, either by letter or in your next issue of HALL'S JOURNAL OF HEALTH.

Yours Respectfully,

E. C. FAITONLE.

On receipt of the foregoing communication, a member of our editorial staff, sought an interview with the Doctors Densmore, who enjoin it upon many of their patients to adopt and rigidly adhere to a vegetarian diet as one of the indispensable means of cure. Mrs. Doctor Densmore was found in their well appointed business office, at 58 West 55th st., and willingly responded to our inquiries, after the following manner:

"Food may be divided into three great classes: The *carbonaceous*, or that which gives heat and vitality to the body; the *nitrogenous*, or that which nourishes the muscles and muscular effort; and the *salts* (of which phosphorous is the more important, and may be classed as the *phosphatic*) which nourish the brain and nervous system, and support all mental effort.

"Physiologists affirm that there are some fourteen to sixteen elements requisite to properly nourish the human body, and wheat not only contains within itself all these elements, but contains them in about the requisite proportions. It has been ascertained that we need nearly five times as much carbonaceous food as nitrogenous, while the amount of salts and phosphatic elements form a small percentage.

"Bread made from the entire wheat, theoretically, is an all sufficient food not only for the development of the muscles and the support of all muscular effort, but also an adequate nourishment for all other departments of the animal organism.

"Is there any scientific proof that grains are a sufficient food for a laboring man?"

"Felix Oswald, M. D., a very brilliant hygienist and reform writer, instances the case of a Polander being sent to a Russian prison at the age of twenty-two. This Polander was allowed two pounds of rye bread and a jug of water per day, with absolutely no other food during the entire year except the week of the anniversary of the crime for which he had been imprisoned, and that week he was allowed one pound of bread per day. This man was pardoned and released at the age of ninety. A life of sixty-eight years on such a diet is surely a scientific proof of its adequacy, especially when you consider the unsanitary conditions, bad air, lack of exercise, etc. to which all prisoners are subject."

It is well known, that whole races of men subsist upon a non-animal diet. A large portion of the Chinese nation subsists largely upon rice, and the millions of India are debarred from meat eating because of their religious tenets. The peasantry of Europe are practically vegetarians. Among the oat cake eating Scotch, the cabbage soup and rye bread eating peasants of northern Russia, the maize and macaroni eating Italians, and the boiled wheat and fruit eating laborers of Constantinople, there are found multitudes of able bodied men and women who are capable of performing Herculean feats of labor, and who are largely exempt from the ordinary diseases of modern civilization."

"What do you consider man's natural diet?"

"Undoubtedly, fruit and nuts. For this reason I suspect that cereals and fruits alone will never prove a perfectly adequate diet, for the reason that nuts have a large percentage of free oil, which is substantially lacking in an exclusively bread and fruit diet. It is my opinion that this element, lacking in cereals and fruits, is indispensable; and that man in former periods, having been deprived of the nuts, which with fruit constitute his natural diet, still longed for the oils which were needed in his system, and that this is one of the reasons for the universal craving for fat meats, butter, cheese, salad oils, and the like. The fact that nuts are not only rich in oils but also abound in nitrogenous elements, will be a surprise to many. A pound of peanuts contains more than double the amount of nitrogenous element found in a pound of ordinary wheat, and is quite equal to that found in a pound of dried beans or peas. Man most nearly resembles the anthropoid ape structurally anatomically and physiologically. These animals are found to subsist in their native forests upon fruits and nuts, and are a wonder of strength and vigor."

"Do you recommend patients who have been accustomed to a meat diet to change all at once to bread and fruit, excluding all animal food?"

"I do not. We have found in quite an extended practice, with chronic diseases, that patients who have been much weakened and emaciated have uniformly received surprising benefits from an uninterrupted use of bread made from the entire wheat broken in milk. This diet is usually recommended for a period of some months and has sometimes been followed by our patients for years with marked benefit."

"Why is it then that people accustomed to meat, lose strength as soon as they stop eating it?"

"A mistake is often made in counselling a too abrupt change. If one is young, or has great vigor, and the powers of digestion and assimilation have not been too much weakened by meat eating and other unnatural foods, and the necessary quantity of cereal foods can be easily digested and assimilated, such a person can be advantageously put upon whole wheat bread and fruit and nuts at once, and all will go well. But many persons have so long depended upon meat and animal foods for a large share of their nourishment, that their digestive organs have become weakened; and if such people are persuaded abruptly to change to brown bread and fruit, they will be quite likely to suffer from flatulence, indigestion, etc., and, what is worse, their weakened stomachs will not have the necessary vigor to abstract the needed elements, from cereal foods, and so they will be likely to suffer from lack of nourishment. We have found that such persons thrive much better for a time on bread and milk than on bread and fruit. This is because milk is much more easily digested and assimilated by weakened stomachs than too much bread; at the same time we do not regard milk as a natural or desirable food, but as a most invaluable crutch on which the meat enfeebled victim may lean in passing from the usual diet of civilization to a more natural diet. One, two, and even three years of bread with milk will yield the most satisfactory results, where an abrupt change to bread and fruit would result in emaciation, weakness, discouragement, and their eventual abandonment

"The favor which you give to milk seems a little strange, when one considers the recent much talked of verdict of some five hundred scientific experts in Paris, that milk causes tuberculosis."

"A few years since it was very commonly affirmed by so called scientific physicians that cancer is often the result of eating too freely of tomatoes. No experiments were made to ascertain wherein tomatoes are injurious, and when it is admitted that these cancer patients use not only tomatoes, but everything else of which modern diet is composed, to assume that the disease is attributible to tomatoes, is absurd. That cows have a kind of consumption in isolated instances, and that disease germs are sometimes found in milk, is undeniable; but the absurdity of discarding milk, and allowing the free use of butter, which is made from the same milk, is easily seen.

"Too much cannot be said against using milk, which is properly a food, as a drink, and furthermore, it ought not to be used as commonly done, where the person has already had an abundant supply of other foods."

"Then, in your view, the matter of diet forms a most important measure, both in the prevention and cure of disease?"

"Our favorite expression is that the diseases of modern life bear the same relation to food that *delirium tremens* does to whiskey. It is well known that if one afflicted with *mania a potu* will discontinue the use of alcoholic stimulants, using only water for drink, the disease will be cured. We maintain, that if people suffering from the usual diseases of civilization will at once abstain from the familiar diet, and learn to eat wholesome foods, the diseases will take unto themselves wings. Sir Henry Thompson, perhaps the most celebrated physician of England, and one, let me say, who is not only a 'regular' Allopath in good standing, but is not suspected of fraternizing with cranks or radicals in any way, affirms that more than one-half of the diseases of modern life are caused by easily avoidable errors in diet. We go further than Sir Henry, and affirm that more than ninety per cent of all the diseases for which people call a doctor are of this class."

---

We next called upon Mrs. George at 251 West 34th st., who conducts a sanitarium, wherein medicated baths and vegetable diet constitute the leading features. Mrs. George went over pretty much the same ground as Dr. Densmore, and in much the same way. She said: "It is about a year ago that I adopted at my table the vegetable mode of living, and have found it to supply all the demands of the system, in a much more satisfactory and healthful way, than the ordinary meat foods, and I am now convinced that meat does not nourish the body. I have substituted in its place pearled oats, broken or cracked wheat, granulated wheat and barley, rice and Indian meal. I make no use of bolted wheat flour, but use instead, the whole wheat flour. Of the commoner vegetables, I give cabbage and onions the preference. Fruits and nuts are excellent in their way, and so are well boiled eggs. I have wholly discarded tea and coffee, and in their place advise a cup of warm almost hot water a half hour or so before meals."

"Do you consider such a course of living adapted to a hard working man?" "Certainly I do, whether he work with his hands or his brain."

